# Multiplex ARMS PCR to Detect 8 Common Mutations of ATP7B Gene in Patients With Wilson Disease

**DOI:** 10.5812/hepatmon.8375

**Published:** 2013-05-16

**Authors:** Hassan Dastsooz, Mohammad Hadi Imanieh, Seyed Mohsen Dehghani, Mahmood Haghighat, Maryam Moini, Majid Fardaei

**Affiliations:** 1 Department of Medical Genetics, Shiraz University of Medical Sciences, Shiraz, IR Iran; 2 Department of Molecular Medicine, Shiraz University of Medical Sciences, Shiraz, IR Iran; 3 Shiraz Transplant Research Center, Gastroenterohepatology Research Center, Namazi Teaching Hospital, Shiraz University of Medical Sciences, Shiraz, IR Iran; 4 Department of Internal Medicine, Gastroenterology and Hepatology Research Center, Shiraz University of Medical Sciences, Shiraz, IR Iran; 5 Stem Cell and Transgenic Technology Research Center, Shiraz University of Medical Sciences, Shiraz, IR Iran

**Keywords:** Multiplex Polymerase Chain Reaction, Hepatolenticular Degeneration, Iran

## Abstract

**Background:**

Wilson disease is a rare disorder of copper metabolism due to mutation in ATP7B gene. Proper counseling of patients with Wilson disease, and their families necessitates finding mutation in ATP7B gene. Finding mutations in ATP7B gene with 21 exons, and more than 500 mutations is expensive and time-consuming.

**Objectives:**

The aim of this study was to provide a simple multiplex amplification refractory mutation system PCR (M-ARMS-PCR) for screening eight common mutations in ATP7B gene.

**Patients and Methods:**

Two sets of ARMS mutant and normal specific primer pairs were designed for genotyping of p.R778L, p.R969Q, p.H1069Q, and p.3400delC mutations as Set 1 and p.W779G, c.3061-1G > A, p.I1102T, and p.N1270S mutations as Set 2. The Multiplex ARMS assay was then subsequently tested in 65 patients with Wilson disease with known and unknown ATP7B mutations.

**Results:**

Using these two sets, we identified H1069Q mutation in four patients, c.2335T > G mutation in three, c.3061-1G > A splice site mutation in five, c.3305T > C mutation in one, and c.3809A > G mutation in two patients.

**Conclusions:**

The Multiplex ARMS assay used in this study can be an efficient, reliable, and cost effective method as a primary screen for patients with Wilson disease.

## 1. Background

Wilson disease (WD) (OMIM 277900) is a rare autosomal recessive disorder, characterized by decreased serum ceruloplasmin concentration, increased levels of 24 h urinary copper excretion, hepatic manifestations, neurological manifestations, and the presence of KF (Kayser-Fleischer) rings (1-5). WD in homozygous state occurs from 1 in 30,000 to 100,000 (2), and it is expected that one in 90 individuals would be heterozygous carrier for this disease (6). Historically, diagnosis is based on the laboratory and clinical findings; however, a molecular approach is required to provide an unambiguous diagnosis of the patients and their families (7, 8). WD is caused by different mutations in ATP7B gene (Ref Seq Gene: NG_008806.1) which encodes a copper-transporting p-type 2 ATPase protein (7, 9). To date, over 500 mutations have been reported that are distributed across the 21 ATP7B exons, exon-intron boundaries, and promoter region. (www.wilsondisease.med.ualberta.ca/search3.asp). Therefore, mutation screening for WD is complicated.

## 2. Objectives

The purpose of this study was to provide a more cost-effective and rapid approach of mutation screening for patients with WD. For this purpose, a multiplex amplification refractory mutation system (M-ARMS) was designed to detect 8 mutations in the ATP7B gene. These mutations were selected on the basis of their frequency in several nations, and also in the south of Iran.

## 3. Patients and Methods

### 3.1. Patients and DNA Samples

Samples consisted of patients with WD referring to Namazi Gastroenterology Center between 2010 and 2012. Diagnosis was made on the basis of an elevated 24 h urinary copper excretion, or an increased liver copper concentration, low level of serum ceruloplasmin, clinical features such as liver disease, neuropsychiatric disease, and the presence of KF rings. All the patients gave written informed consent before undergoing DNA test for ATP7B mutation analysis according to the ethics committee in Shiraz University of Medical Sciences. Three ml of the peripheral venous blood samples were collected into EDTA tubes from 65 patients with WD. The blood samples were stored at -20ºC until use.DNA was extracted by AccuPrep® Genomic DNA Extraction Kit (Bioneer, Korea) according to the manufacturer's recommendations. The DNA quantity was measured by NanoDrop (ND1000, USA), and the extracted DNA was stored at -20ºC until use.

### 3.2. M-ARMS Primer Design

Two sets of mutant-specific primers (Set 1 and 2) were designed to generate four mutant amplicons, and two control amplicons in each set. Set 1 contained primers for the detection of the most common mutations of ATP7B gene, including c.2333G > T (p.R778L), c.2906G > A (p. R969Q), c.3207C > A (p.H1069Q), and c.3400delC located in exons 8, 13, 14, and 15, respectively, and also two internal control amplicons from the two intronic sequences in ATP7B gene, lower and upper controls (Table 1). Set 2 contained primers for four mutations identified by DHPLC analysis in patients with WD from the south of Iran; some of the mutations were more common (10), including c.2335T > G (p.W779G), c.3061-1G > A, c.3305T > C (p.I1102T), and c.3809A > G (p.N1270S), and two internal controls were the same as Set1 (Table 2). To discriminate hetero- and homozygote variants, two sets of these primer pairs were also designed to create normal and control amplicons, Set 1A and 2A. These sets contained primers specific for normal DNA which was different from the Sets 1 and 2 at their 3′ end nucleotides (Tables 1 and 2). In this case, a heterozygote mutation would be amplified with both normal and mutant primers, but a homozygote mutation would be only amplified with the primer specific for the mutation. These primer pairs were designed, and evaluated on the basis of the ATP7B genomic sequence (GenBank accession no: NG_008806) using several websites and bioinformatics softwares such as Oligocalc, Primer3, Primer-BLAST, NCBI-BLAST, PRIMER Biosoft, and PrimerPlex 2.50. The specificity of the ARMS PCR primers was enhanced by the introduction of an additional mismatch at the second, third, or fourth nucleotide from the 3' end of the primer. In the primer in which the 3' terminal mismatch was strong (C–C, G–A, and A–A), a weak secondary mismatch was introduced, and if the 3' terminal mismatch was weak, a strong secondary mismatch was selected (11). The M-ARMS was standardized on DNA samples with four known mutations of Set 2 (c.2335T > G, c.3061-1G > A, c.3809A>G, c.3305T > C), and one known mutation of Set 1 (c.3207C > A).The Multiplex ARMS assay was then subsequently tested in 65 patients with Wilson disease with 15 known, and 50 unknown ATP7B mutations.

**Table 1.** Set 1 and 1A of M-ARMS a

Mutation	PCR product (bp)	Exon	Sequences (5' > 3')
**R778L (c.2333G > T)**	261 (mutant primer)	8	F: GCAGCCTTCACTGTCCTTGTCTT
	260 (normal primer)		R: CTTTGCCAAGTGTTCCAGCCTC*A*(M)^b^
			R:TTTGCCAAGTGTTCCAGCCTCC(N)^b^
**R969Q (c.2906G > A)**	212 (mutant primer)	13	F: CATCTCCCAGACAGAGGTGATCATAC*A*(M)
	209 (normal primer)		F: CTCCCAGACAGAGGTGATCATCCG (N)
			R: CAGGATGGGGAAAGCCGTGCTA
**H1069Q (c.3207C > A)**	391	14	F: TGCGGAGGCCAGCAGTGAATA*A*(M)
	(both primers)		R: TGCGGAGGCCAGCAGTGAATAC (N)
			R: TGTCAAAGCACTGAGTTTCCAGACTG
**c.3400delC**	492 (mutant primer)	15	F: TCCTTTCCAGTCGGTAACCTGTTCA
	491 (normal primer)		R: AGCCAGCAATACCTTTTTCTGCGTA (M)
			R: CCAGCAATACCTTTTTCTGCGGGAA(N)
**Lower control **	153		F: AGTGGTCGTTTTAGCAGCAACAGAG
			R: GTGTTCATGTTACTGGGCCATCTCC
**Upper control**	585		F: CCACCGTCAGAGGAAGGAGAATTTC
			R: CTAGGTCAATGAAGAAGACCCTGTACAC

^a^Mutant-specific nucleotide is shown in bold and italic letter. Deliberate mismatch is underlined

^b^M, mutant-specific primers in Set 1; N, normal-specific primers in Set 1A

**Table 2.** Set 2 and 2A of M-ARMS PCR a

Mutation	PCR product (bp)	Exon	Sequences (5' > 3')
**W779G (c.2335T > G)**	294 (both primers)	8	F: TCGCTCATTGAACTCTCCTCCCT
			R:ACCTTTGCCAAGTGTTCCAGAC*C* (M)^b^
			R: ACCTTTGCCAAGTGTTCCAGTCA (N)^b^
**I1102T (c.3305T > C) **	415 (both primers)	15	F: GCAGTGCCAGGCTGTGCAA*C* (M)
			F: GCAGTGCCAGGCTGTGCAAT (N)
			R: CTCTGTAGCTTATGAGAAGCAAGACCG
**c.3061-1G > A**	330 (both primers)	14	F: CAGTGAGTTGTGGTTGTTTTTGCCA*A*(M)
			F: CAGTGAGTTGTGGTTGTTTTTGCCAG (N)
			R: CTCTAAGTGGTTTTCCAGACCACACAG
**N1270S (c.3809A > G)**	507 (both primers)	18	F: CCATGGTGGGGGATGGGGTAA*G*(M)
			F: CCATGGTGGGGGATGGGGTAAA (N)
			R: GTTTCAGGTCCTCTCCACAGTTTCTC
**Lower control**	153		F: AGTGGTCGTTTTAGCAGCAACAGAG
			R: GTGTTCATGTTACTGGGCCATCTCC
**Upper control**	585		F: CCACCGTCAGAGGAAGGAGAATTTC
			R:CTAGGTCAATGAAGAAGACCCTGTACAC

^a^Mutant-specific nucleotide is shown in bold and italic letter. Deliberate mismatch is underlined

^b^M, mutant-specific primers in Set 1; N, normal-specific primers in Set 1A

### 3.3. Multiplex ARMS PCR

The PCR reaction was performed with QIAGEN Multiplex PCR master mix kit (QIAGEN, Germany) using Eppendorf Mastercycler Gradient (Germany). The Multiplex PCR kit contained preoptimized concentrations of HotStar Taq DNA Polymerase, and MgCl_2_, plus dNTPs, and a PCR buffer which allowed efficient primer annealing and extension. To begin with, 10x primer mixes (containing each primer at 2 µM) were prepared according to the QIAGEN Multiplex PCR kit. Multiplex PCR reactions were then prepared in 50µl containing 25 µl of 2x QIAGEN Multiplex PCR Master Mix, 5 µl of 10x primer mix, 50-100 ng DNA, and enough RNase-free water. Finally, the PCR reaction was performed using Eppendorf Mastercycler Gradient according to the following protocol: 95°C for 15 min, then 35 cycles at 94°C for 30s, annealing temperature for 90s (Set 1: 67°C, Set 1A: 62°C, Set 2: 64°C, Set 2A: 65°C), 72°C for 45s followed by a final extension step at 72°C for 30 min. Following amplification, 10 µl of the PCR reaction was electrophoresed along with a 100-bp ladder (Vivantis) on a 2% agarose gel and visualized under the UV lightbox (GBOX, SYNGENE, UK).

## 4. Results

In the present study, the M-ARMS were designed to identify the eight common mutations of ATP7B gene. The ATP7B ARMS assay consisted of two separate PCR sets, and each containing primer for four different mutations. To confirm PCR success, two pairs of control primers were included in each set. The M-ARMS was standardized on DNA samples with four known mutations of Set 2, and one known mutation of Set 1. The method was subsequently tested on 67 samples with known and unknown ATP7B gene mutations.

### 4.1. Set 1 and Set 1A of Multiplex PCR

**Figure 1.**
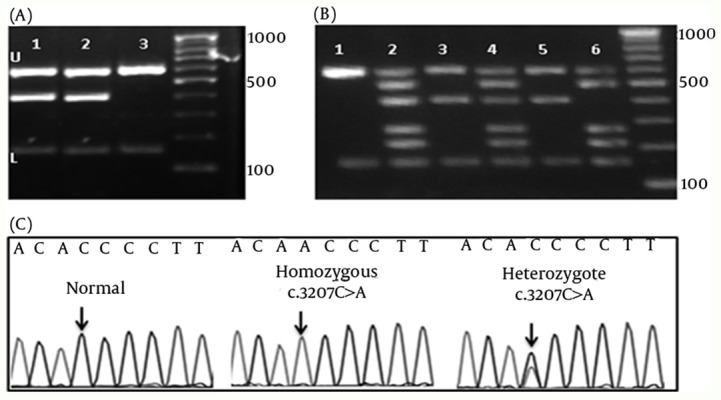
Chromatograms and Gel Electrophoresis Images of the Mutation, and the DNA Bands of Set 1 and 1A (A) Gel electrophoresis of Set 1, U: upper and L: lower controls are depicted for all lines. 1 and 2 show the band for the c.3207C > A mutation.3: Normal DNA which shows only the control amplicons. 100bp DNA ladder is depicted in left. (B) Gel electrophoresis of the DNA bands of the hetero- and homozygote c.3207C > A mutation. 1 and 2 shows the normal control DNA using Set 2 and 2A, respectively. 3 and 4 show the heterozygote c.3207C > A mutation. 5 and 6 show patients with the homozygote c.3207C > A mutation. 100bp DNA ladder is depicted in left. (C) Chromatograms of main mutation detected in Set 1.

Using M-ARMS for Set 1, c.3207C>A, mutation was detected in four of 65 patients with WD (cases 2, 4, 11, and 18) (Figure 1-A). Presence of this mutation was confirmed by direct DNA sequencing. Set 1A was also performed for patients with the compound heterozygote and homozygote c.3207C > A mutation to confirm the efficiency of this method for genotyping (Figure 1-B). In case 18 with the heterozygote c.3207C > A mutation, Set 1 of M-ARMS PCR showed only the control bands (153 bp, and 585 bp), and the related mutant band (391 bp), but Set 1A showed all bands expected to be detected in the presence of normal allele (Figure 1-B, third and fourth wells). Patient with the homozygote c.3207C > A mutation (case 4) showed the control bands (153 bp, and 585 bp), and the related mutant band (391 bp) with Set 1, and all bands except the band for this mutation with Set 1A (Figure 1-B, fifth and sixth wells). Normal DNA showed only control amplicons using Set 1 (Figure 1-B, first well), but all normal amplicons were amplified using Set 1A (Figure 1-B, second well). Sequencing graph for this mutation was shown in figure 1-C. These observations confirmed that case 18 was a compound heterozygote.

### 4.2. Set 2 and 2A of Multiplex PCR

**Figure 2.**
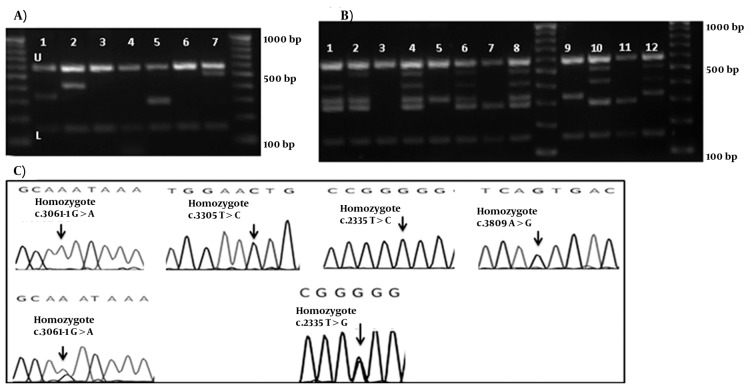
Chromatograms and gel Electrophoresis Images of the Mutation and the DNA Bands of Set 2 and 2A (A) Gel electrophoresis of Set 1, U: upper and L: lower controls are depicted for all lines. 3rd, 4th, and 6th wells show bands for the normal DNA. 100bp DNA ladder is depicted in right and left. Using Mix Set 2, Samples in 1st, 2nd, 5th, and 7th wells show the band for c.3061-1G > A, c.3305T > C, c.2335T > G, and c.3809A > G mutations, respectively. (B) Gel electrophoresis of the DNA bands of the hetero- and homozygote c.2335T > G, and c.3061-1G > A mutations. 1: the normal DNA with Set 2A. 2 and 3: heterozygotec.3061-1G > A mutation. 4 and 5: heterozygote c.2335T > G mutation; 6 and 7: homozygote c.3061-1G > A mutation. 8 and 9: homozygote c.2335T > G. 100bp DNA ladder is depicted in middle and left. (C) Chromatograms of main mutation detected in Set 1.

M-ARMS PCR of Set 2 was performed for 65 patients with Wilson disease. Similarly for Set 2, all samples showed both control bands, and bands from c.2335T > G mutation in three cases, c.3061-1G > A splice site mutation in four cases, c.3305T > C mutation in one case, and c.3809A > G mutation in two cases (examples of cases with these mutations are shown in Figure 2-A. first, 2nd, 5th, and 7th wells). Distribution of ATP7B mutations (n = 65) detected by Set 1 and 2 of multiplex ARMS, and sequencing technique are given in Table 3. No band other than the internal control was amplified in the DNA without these mutations (Figure 2-A. 3rd, 4th, and 6th wells). The assay was designated as a fail try if both PCR control bands were not amplified, and the M-ARMS assay was repeated. To confirm the efficiency of this method for genotyping, Set 2A was performed for c.2335T > G mutation in samples with a homozygote and a heterozygote (a sample from mother of this homozygous patient) condition. Set 2A was also performed for c.3061-1G > A mutation in patients with a homozygote, and a compound heterozygote condition. Using Set 2 of M-ARMS assay, patients with the homozygote c.2335T > G mutation (case 3), and c.3061-1G > A mutation (case 8) showed the control bands (153 bp, and 585 bp), and the related mutant bands (294 bp for c.2335T > G and 330 bp for c.3061-1G > A), and using Set 2A all bands except the bands for these mutations were amplified (Figure 2-B, 8th, and 9th wells for the case 3; and 6th and 7th wells for case 8).

In the mother of case 3 with the heterozygote c.2335T > G mutation, Set 2 of M-ARMS PCR showed only the control bands (153 bp, and 585 bp), and the mutant band (294 bp), but Set 2A showed all bands expected to be detected in the presence of the normal allele (Figure 2-B, 4th and 5th wells). In case 17 with the heterozygote c.3061-1G > A splice site mutation, Set 2 of M-ARMSPCR showed only the control bands (153 bp, and 585 bp), and mutant band (330 bp), but Set 2A showed all bands expected to be detected in the presence of the normal allele (Figure 2-B, 2nd and 3rd wells). Normal DNA showed all normal amplicons using Set 2A (Figure 2-B, first well); these observations confirmed that case 17, and the mother of case 3 had the heterozygote mutation. Sequencing graph for these mutations were shown in figure 2-C.

**Table 3.** The Distribution of ATP7B Mutations (n=65) Detected by Set 1 and 2 of Multiplex ARMS and Sequencing Technique

Genotype			M.ARMS in Set 1				Patient
Sequencing	C.H^a^	H^a^	c.3400delC Exon 15	c.3207C > A Exon 14	c.2906G > AExon 13	c.2333G > T^4^Exon 8	
**c.3207C > A**	-	+	-	+	-		Case 2
**c.3207C > A**	-	+	-	+	-	-	Case 4
**c.3207C > A**	-	+	-	+	-	-	Case 11
**c.3207C > A**	+	-	-	+	-	-	Case 18
**Genotype**			**M.ARMS in Set 2**				**Patient**
**Sequencing**	**C. H**	**H **	**c.3809A > ** **GExon18**	**c.3305T > ** **CExon15**	**c.3061-1G > ** **AExon14**	**c.2335T > ** **GExon8**	
**c.3809A > G**	-	+	+	-	-	-	Case 1
**c.2335T > G**	-	+	-	-	-	+	Case 3
**c.2335T > G**	-	+	-	-	-	+	Case 5
**c.3061-1G > A**	-	+	-	-	+	-	Case 6
**c.3061-1G > A**	-	+	-	-	+	-	Case 7
**c.3061-1G > A**	-	+	-	-	+	-	Case 8
**c.3305T > C**	-	+	-	+	-	-	Case 10
**c.2335T > G**	-	+	-	-	-	+	Case 12
**c.3809A > G**	-	+	+	-	-	-	Case 14
**c.3061-1G > A**	-	+	-	-	+	-	Case 15
**c.3061-1G > A**	+	-	-	-	+	-	Case 17

^a^H, homozygote, C.H, compound heterozygote

## 5. Discussion

Several mutations of ATP7B gene have been reported with high frequency in different nations. The common mutations of ATP7B gene in patients with WD originating from Eastern, Northern, and Central European are H1069Q (at exon 14) (12-15), R969Q (at exon 13) (12-15), and 3400delC (at exon15) (12, 13, 15, 16). R778L (at exon8) is the most common mutation in the East Asian population (17, 18) (database maintained at the University of Alberta: www.wilsondisease.med.ualberta.ca/search3.asp). Mutation used for these two sets were also detected in Iranian population (19). These observations suggest that exons 8, 13, 14, and 15 may be four hot spots for the identification of ATP7B mutations in several nations. In specific populations with high frequencies of the most common mutations, mutation screening approaches which detect such mutations have been described. To investigate several common mutations, M-ARMS PCR can be very useful and cost-effective. The purpose of this study was to determine the efficiency of an M-ARMS assay for the identification of the most common ATP7B gene mutations identified in many populations. To have successful results of ARMS assay, the melting and annealing temperature of all primers in each set was very similar, and also all primer pairs had similar concentrations in the final mix. Overall, in this study, the M-ARMS PCR detected 5 different mutations in 15 cases. No false-negative or false-positive results were obtained using this M-ARMS assay. Exact identification of the mutations in the samples using ARMS assay confirmed the high accuracy of this assay. To detect common mutations of ATP7B mutations in the south of Iran, a procedure such as the M-ARMS is efficient and practical, because several ATP7B mutations can be simultaneously screened. ARMS procedure which detects several mutations of a given gene in different primer Multiplexes is available and used in clinical diagnoses (20-22). Therefore it would be possible to increase the detection of ATP7B gene mutations in the south of Iran by adding additional appropriate primer Multiplexes. The M-ARMS protocol described in this study has provided an accurate, rapid, inexpensive, and direct molecular testing for WD. Depending upon current and future studies, it would be possible to optimize and establish the M-ARMS-PCR for other common mutations of ATP7B for the Southern Iranian population.
